# Responsibility, prudence and health promotion

**DOI:** 10.1093/pubmed/fdy113

**Published:** 2018-07-11

**Authors:** R C H Brown, H Maslen, J Savulescu

**Affiliations:** Oxford Uehiro Centre for Practical Ethics, University of Oxford, Oxford, UK

**Keywords:** prudence and health promotion, responsibility

## Abstract

This article considers the role of responsibility in public health promotion. Efforts to tackle non-communicable diseases which focus on changing individual behaviour and reducing risk factor exposure sometimes invoke individual responsibility for adopting healthy lifestyles. We provide a critical discussion of this tendency. First, we outline some key distinctions in the philosophical literature on responsibility, and indicate how responsibility is incorporated into health promotion policies in the UK. We argue that the use of some forms of responsibility in health promotion is inappropriate. We present an alternative approach to understanding how individuals can ‘take responsibility’ for their health, based on the concept of prudence (i.e. acting in one’s interests). In this discussion, we do not prescribe or proscribe specific health promotion policies. Rather, we encourage public health professionals to consider how underlying assumptions (in this case, relating to responsibility) can shape health promotion policy, and how alternative framings (such as a shift from encouraging individual responsibility to facilitating prudence) may justify different kinds of action, for instance, shaping environments to make healthy behaviours easier, rather than using education as a tool to encourage responsible behaviour.

## Introduction

One of the biggest challenges facing modern healthcare systems is the burden of non-communicable disease (NCD). Diseases related to behavioural risk factors including diet, physical inactivity, smoking and alcohol consumption are the leading cause of death globally. Numerous World Health Organization (WHO) member states have pledged to tackle NCDs, agreeing to global targets to reduce premature mortality from NCDs by one-third by 2030.^1^

Since individual lifestyles partly determine risk factor exposure, it seems initially plausible that individuals can ‘take responsibility’ for their health (e.g. by eating healthily, exercising more, smoking and drinking less), and that emphasizing responsibility should inform strategies to tackle NCDs. Advocates of responsibility-based approaches (which often include libertarians and those in favour of minimal industry regulation) argue that it is both fair and respectful to recognize the role individuals can play in maintaining their health by ensuring public health interventions are responsibility-sensitive.^2–5^ Whilst highlighting the ‘empowering’ potential of responsibility, such advocates simultaneously criticize the infantalizing alternative of the ‘nanny state’. Sceptics argue that incorporating assumptions about individual responsibility into health promotion policies is misguided, impractical or unjust, and highlight the importance of the social and built environment in determining behaviour, risk factor exposure and health.^6–8^ Attitudes towards individual responsibility tend to align with broad political leanings, such that responsibility advocates tend to be more right-leaning, favouring individualism, whilst responsibility sceptics tend to be more left-leaning, favouring collectivist approaches to political and social organization.

In this article, we first outline how responsibility has been described by philosophers. We introduce some distinctions between different kinds of responsibility and what the identification of someone as ‘responsible’ requires and entails. We then give examples of how policies and interventions to promote health incorporate assumptions about responsibility. Next, we highlight some problems with incorporating responsibility into health promotion, arguing that it rests on dubious assumptions about individual control over behaviour. We propose an alternative way of thinking about the role of individuals in health promotion, using the concept of prudence to capture how health promoters can facilitate healthy behaviour.

## What is ‘responsibility’?

Philosophers distinguish between ‘causal’ and ‘moral’ responsibility, relating respectively to the causal and moral roles of an actor in bringing about some consequence. Since morality is the sort of thing that only attaches to agents, non-agents (dogs, rocks, weather, etc.) can be causally responsible but not morally responsible. Sometimes, people act in ways that bypass or oppose their agency: someone having a seizure may thrash around and injure another, or someone under the threat of violence may commit a crime. In these cases, the person’s diminished agency may serve to void or mitigate her moral responsibility, even though she remains causally responsible. Allocation of praise or blame may be appropriate following the identification of moral responsibility.

Discussion of the distribution of benefits and burdens, rewards and punishments, praise and blame, typically draws upon ideas of moral responsibility (Who ‘deserves’ the credit for inventing penicillin?; Who ‘should pay’ compensation to victims of medical negligence?). Causal responsibility is often a necessary but insufficient condition for moral responsibility. Two conditions for moral responsibility are generally identified: ‘epistemic’ and ‘control’ conditions. The epistemic condition requires that the agent was able to foresee the likely consequences of her actions. The control condition requires that the agent was able to control her actions. The person having a seizure was not able to foresee the timing of her seizure, nor control it (and so fulfils neither condition for moral responsibility); the person who commits a crime under duress likely does fulfil the epistemic condition, although her control over her actions was compromised.

Finally, we might distinguish between two different sorts of moral responsibility: ‘attributability’ and ‘accountability’.^9^ These recognize differences in the degree to which the epistemic and control conditions are satisfied, beyond the binary identification of moral responsibility as either present or absent. Accountability is more robust, and indicates that it is appropriate to hold the individual accountable for her actions, for instance, through punishment. Attributability is less demanding, requiring only that the particular actions be attributable to the agent (in the sense that she can take ownership of them). Attributability may be relevant where people behave wrongfully, but where contextual factors or wider circumstances play a significant role in determining their behaviour.

## Responsibility in health promotion

Assumptions about responsibility can be tacitly incorporated into health promotion policies as well as play an explicit role. ‘Robustly responsibilising’ policies involve ensuring that any negative health consequences of an individual’s behaviour are strictly experienced by that individual, sometimes with additional rewards or punishments. These policies hold individuals accountable. Such policies would include, for example, restricting treatment for smoking-related diseases, or rewarding people of a healthy weight with tax incentives.

In the UK, there are few examples of health promotion policies which are robustly responsibilising. A basic principle of UK healthcare provision is that it should be provided according to need and capacity to benefit, and considerations such as one’s responsibility for becoming sick ought not to factor into entitlement to treatment. Along with ensuring that smokers receive treatment for smoking-related disease, this also ensures that those who are obese receive treatment for type II diabetes, and that recovering alcoholics are given equal access to liver transplants. It is arguable that some policies, such as delaying surgeries or denying fertility treatment for obese people and smokers, are effectively robustly responsibilising, even if the justification given for them denies that this is so.^10,11^

Policies can also be ‘weakly responsibilising’. These policies attribute responsibility. The use of information provision and educational strategies suggests that individual behaviour change is achievable through empowerment and healthy choices. An extension of these efforts is the use of social marketing campaigns. These have become popular tools of health promotion, exploiting techniques from the commercial sector to design, target and deliver information about health (including behavioural risk factors). Campaigns such as ‘Change4Life’, ‘Stoptober’ and ‘5ADay’ emphasize the power of individuals to make healthy changes to their behaviour. Such campaigns signal that the responsibility for reducing risk factor exposure and improving health lies with individuals themselves: once people are informed about the risks to their health of high calorie diets, lack of physical activity, smoking and drinking, then it is up to them to change their behaviour. Information and education can be described as weakly responsibilising because they identify people as responsible for making healthy choices, without making treatment conditional upon doing so.

Policy documents often use the language of fostering empowerment, facilitating choice and the need to create responsible individuals. For instance, the NHS Constitution for England instructs the reader to ‘recognize that you can make a significant contribution to your own, and your family’s, good health and wellbeing, and take personal responsibility for it’.^12^ In the ‘Five Year Forward View’ the NHS leadership lays out its plans for establishing preventive measures to tackle NCDs, asserting that ‘there is broad consensus on what [the] future needs to be. It is a future that empowers patients to take much more control over their own care and treatment’.^13^ In a recent report, Public Health England describes how: ‘[behavioural] risk factors reflect the choices that we all make’, adding the qualifier: ‘and the ways that our choices are shaped by the social circumstances of our lives, such as employment, education, housing, income and relationships’.^14^ The language of these policy statements indicates an explicit role for responsibility in changing health-related behaviour, only occasionally acknowledging the consensus emerging across a range of social and behavioural science research that physical, social and economic environments are central to (changing) behaviour.^15,16^

## Doubts about responsibility

If people are considered morally responsible for their behaviour—in particular, if they are deemed accountable—then it may seem appropriate for them to bear the full cost of any negative health effects they suffer as a result. There are, however, a number of reasons why such an approach should be avoided. First, it is not clear that people are individually accountable for their behaviour in the required way. For instance, researchers now emphasize how choices (particularly habitual behaviours) are shaped by environmental, social and political factors and often resistant to information provision.^17–19^ In combination with findings from research into the social determinants of health,^20^ this suggests people may lack the individual control necessary to be considered morally responsible for failing to change their behaviour. This has led to criticism of the use of responsibility in health promotion, either as a tool, an output or an assumption.^21^

Second, even if there were evidence that at least some people, some of the time, have sufficient control over their behaviour to be considered accountable, this alone would not justify robustly responsibilising interventions. It will be difficult to determine who does and does not have sufficient control, and thus, who is accountable and who is not. Policies which hold people accountable will thus wrongly hold accountable people who, in fact, lacked sufficient control. We propose that it is worse to punish or disadvantage those who lacked the control necessary to be fully morally responsible for their behaviour, than it is to fail to hold accountable those who possessed such control. Thus, we suggest favouring a presumption against holding people accountable for poor health they suffer as a result of engaging in unhealthy habits.

One may think, given our earlier acknowledgment of the relative rarity of robustly responsibilising policies in UK health promotion, that this conclusion has little impact. However, we consider that the presumption against accountability stretches beyond a need to avoid policies that are explicitly punishing or blaming. It requires that the effects of policies must not involve unjust disadvantage, punishment or blame. As we discuss below, we worry that weakly responsibilising policies could result (often unintentionally) in blaming and disadvantage by signalling that people are accountable, and fostering this belief in the public consciousness. This occurs through signalling that self-control is achievable and by appearing to affect fulfilment of the epistemic and control conditions via information provision (which, as mentioned, is often insufficient to change behaviour).

## A role for prudence?

Whilst it is possible to capture the idea that people are responsible for their health-related behaviour via attributability, without claiming that they are also accountable for its consequences, it is hard to see what implications this has for health policy. We suggest a more fruitful approach to conceptualizing the role of the individual in health promotion efforts is captured by the idea of ‘prudence’ (R.C.H. Brown *et al*., unpublished results). Prudence as a technical concept is embedded in the philosophical literature on theories of wellbeing, describing how people can strive to behave in ways that accord with their own interest in their wellbeing, which is partly determined by the things they personally enjoy or desire. Although people will differ in what they enjoy or desire, maintaining health will often be important for the pursuit of certain projects and enjoyment of certain experiences.

Thus, even if the opportunities for people to change their behaviour are limited, it may still be prudent for them to attempt to do so, without rendering them blameworthy for any failure to succeed. The role of health promotion can be seen as vital to supporting prudent behaviour, whilst recognizing the limited control that individuals can wield over their health: if, for most people, their interests are likely to be realized by avoiding NCDs, then public health promoters will be justified in crafting environments that make avoidance of behavioural risk factors easier, for instance, by shrinking portion sizes or creating healthy default options.^22^

Discussing responsibility in terms of accountability and attributability encourages a focus on the moral blameworthiness or praiseworthiness of the individual on the basis of her unhealthy or healthy behaviour. Focusing, instead, on facilitating prudence de-emphasises such backward-looking responsibility. Whilst prudence directs individuals to try to further their interests, it does not demand success. Since changing health-related behaviour is difficult, and many who attempt to discard unhealthy behaviours will fail, no criticism or blame should be attached to this failure. Yet since it will be in many people’s interests to avoid risk factors for NCDs, they still have prudential reasons for attempting to do so (assuming the chances of success are not so small as to render them not worth pursuing).

Health promotion thus has a role in facilitating a kind of prudential responsibility, which one has in relation to one’s own wellbeing: using evidence from the social and behavioural sciences to design environments that make healthy (prudent) behaviours easier. What counts as prudent behaviour will ultimately be determined by features of the individual: what she finds pleasurable, what preferences she holds, the sorts of activities she values, and her hopes and plans for the future. Whilst some accounts of wellbeing emphasize its objective components, most assume sensitivity to individual preferences and circumstances are important, at least in part.^23,24^

For example, regarding alcohol consumption, campaigns can permissibly appeal to the ways in which alcohol might reduce short- or long-term wellbeing—perhaps by affecting sleep, mood, mental clarity or relationships—and provide strategies, support lines or groups, and information about the effects of alcohol on the body. Of course, as with any intervention, it will be vital to discriminate between more and less effective approaches, as indicated by evidence-based research into downstream versus upstream interventions (i.e. those targeting individuals versus those targeting populations).^25^ Such campaigns should not assume that abstinence is prudentially optimal for all people, nor that everyone will have all or any of the possible prudential reasons to cut down. Language must also avoid any implications that drinking is morally objectionable/indicates moral failure in the individual. Whilst we recognize that certain stigmatizing campaigns (e.g. around drink driving) have been highly successful, such an approach is much more controversial in cases where the target behaviour is not clearly morally wrongful (as, for instance, with drinking, smoking or overeating). Policies that assume moral responsibility and desert (e.g. lower priority for liver transplants) would be unjustified.

## Discussion

### Main finding of this study

Policy based on accountability is not only philosophically inconsistent, but misconstrues people’s responsibility in unjust and potentially harmful ways. For instance, social marketing can encourage the view that people should adopt healthy lifestyles: that those engaging in healthy habits behave ‘well’ and those engaging in unhealthy habits behave ‘badly’. This can foster moralization and stigmatization of particular individuals or groups, which is associated with established harms and may have counterproductive effects on health.^26–29^ Any restriction in access to healthcare for those deemed responsible for their disease due to unhealthy behaviour may also exacerbate health (and other) inequalities, since it is typically the poorest who suffer the most from lifestyle-related NCDs. Identifying a role for health promotion as facilitating prudence may capture the duties of states to promote health whilst supporting individuals in pursuing the kinds of lives that are important to them.

### What is already known on this topic?

Behavioural risk factors play a role in NCDs, and responsibility language is often used in policies to tackle unhealthy behaviours. This conflicts with multi-disciplinary research which shows that individual control over behaviour is often limited, with social and environmental factors being more influential. Philosophical understandings of the value and nature of responsibility could helpfully illuminate the role (or lack of role) for individual responsibility in the context of health promotion.

### What this study adds

Our arguments point towards the following approach to health promotion policies and campaigns: on the one hand, health promotion policies should avoid (i) holding people responsible (e.g. allocating or prioritizing medical resources on grounds of individuals’ [apparent] responsibility for their predicament) and (ii) communicating moral evaluation of unhealthy behaviours (e.g. avoid language that implies moral assessment in public campaigns). On the other hand, our claims regarding prudential responsibility provide a justification for the particular kinds of health campaigns that equip individuals with the information or resources to act in line with their interests. Permissible campaigns will appeal only to the sorts of prudential reasons that people have to maintain their health. Such campaigns will also not assume that everyone’s wellbeing will be served in the same way by adopting healthier behaviour. To some extent, the existing systems of patient and public involvement, and the avoidance of paternalism are attempts to incorporate different perspectives into the design of health policies and to protect individuals from intrusive interventions by healthcare providers. The notion of prudence can be used to complement such tools. Aiming at facilitating prudence, rather than allocating moral responsibility, is a more defensible basis for designing ethical approaches to health promotion.

### Limitations of this study

A consideration of the broader effects of any responsibilising policy—both in terms of health and economic outcomes, as well as impacts on autonomy, inequality and so on—will be needed for a full ethical analysis of its use. Our discussion has focused only on issues relating to responsibility, and so cannot provide overall recommendations in relation to specific policies. Further, the nature of prudence makes it a difficult subject to measure compared to concepts with more objective features (such as biomedical health), meaning it is also difficult to assess the success of interventions targeted at supporting prudence. Further investigation into how prudence could be measured and increased could provide fruitful topics for future research.
